# Naming difficulties after thyroid stimulating hormone suppression therapy in patients with differentiated thyroid carcinoma: a prospective cohort study

**DOI:** 10.1007/s12020-019-01943-8

**Published:** 2019-05-05

**Authors:** Shan Jin, Yun-Tian Yang, Wuyuntu Bao, Yinbao Bai, Jing-Wen Ai, Yousheng Liu, Hong Yong

**Affiliations:** 1 Department of General Surgery, Affiliated Hospital of Inner Mongolia Medical University, Hohhot, 010050 Inner Mongolia Autonomous Region China; 2 Departments of Neurology, Affiliated Hospital of Inner Mongolia Medical University, Hohhot, 010050 Inner Mongolia Autonomous Region China; 3 Public Health School of Inner Mongolia Medical University, Hohhot, 010100 Inner Mongolia Autonomous Region China

**Keywords:** Differentiated thyroid carcinoma, TSH suppression therapy, Cognitive impairment

## Abstract

**Background:**

Thyroid stimulating hormone (TSH) suppression therapy after differentiated thyroid carcinoma surgery causes cognitive impairment. However, data on naming difficulties (anomia)—related specific cognitive impairment are lacking.

**Methods:**

A prospective cohort study was conducted, in which, patients with differentiated thyroid carcinoma and benign thyroid nodules were given oral L-T4 therapy after surgery, after meeting the criteria of TSH suppression therapy and thyroxine replacement therapy, respectively, the patients were continually given l-T4 therapy for 6 and 12 months, and then, the neuropsychological test was performed.

**Results:**

Of the 255 subjects, 212 cases (83.13%) completed all the tests, including 33 cases in the normal control group (NC group), 110 cases in the TSH suppression therapy group (TS group), and 69 cases in the thyroxine replacement therapy group (TR group). There was no significant difference in background data among the three groups (*P* > 0.05). The scores of mini-mental state examination, clock drawing test, digit symbol substitution test, personal history, temporal and spatial orientation, digit order relation, visual object recognition, associative learning, and color naming in the TS and TR groups were not significantly different from those in the NC group after 6 and 12 months of L-T4 therapy (*P* > 0.05); the scores of picture recall, visual recall, comprehension memory, and digit span forward in the TS and TR groups were notably lower than those in the NC group (*P* < 0.01); the scores of confrontation naming and listing the names in the TS group were significantly lower than those in the NC and TR groups, and the scores decreased with the prolongation of TSH suppression therapy (*P* < 0.01).

**Conclusion:**

TSH suppression therapy after differentiated thyroid carcinoma surgery could lead to short-term memory impairment, attention impairment, word selection anomia, and depression, of which, word selection anomia was aggravated with the prolongation of TSH suppression therapy. Therefore, we suggested that optimal TSH goals for individual patients must balance the potential benefit of TSH suppression therapy with the possible harm from subclinical hyperthyroidism especially in low risk differentiated thyroid carcinoma patients (ClinicalTrials.gov Protocol Registration System: ClinicalTrials.gov ID NCT0266532, Registered on 21 June 2016).

## Introduction

Normal thyroid function is an important basis for maintaining the best cognitive function. Thyroid hormone affects the expression of genes that play an important role in learning, memory, and synaptic plasticity in the brain, that are all critical for maintaining normal cognitive function [1]. Thyroid hormone plays a significant role in regulating nerve growth, especially in brain regions (e.g., hippocampus), which is closely associated with memory [2]. It has been proved that abnormal thyroid function can lead to cognitive and emotional impairment, including in memory, attention, perception, visuospatial function, executive function, emotional disorders, and even other psychiatric manifestations [3].

Thyroid cancer is a common malignant tumor of endocrine system, and the incidence is annually increasing as well [4]. More than 90% of thyroid cancers are differentiated thyroid cancer (DTC). l-thyroxine (l-T4) suppression therapy after DTC surgery is the standard treatment procedure after thyroid cancer surgery [5]. The level of thyroid-stimulating hormone (TSH) suppression is closely associated with recurrence, metastasis, and cancer-related death of DTC; especially in high-risk DTC patients, increase of serum TSH level can promote the progress of DTC after surgery [6]. However, long-term TSH suppression therapy in DTC patients after surgery can lead to drug-induced subclinical hyperthyroidism. Although TSH suppression therapy is essentially different from primary hyperthyroidism, long-term TSH suppression therapy can cause cognitive and emotional impairment. A number of studies suggested that TSH suppression therapy may lead to cognitive impairment in DTC patients, involving executive function, information processing speed, attention, and it easily leads to emotional, sleep, human communication and other disorders [7–10]. However, some studies suggested that long-term TSH suppression therapy had no significant effect on cognitive function in elderly patients [11]. The above data reflected that TSH suppression therapy after thyroid cancer surgery is associated with impaired cognitive function, while the majority of the research data have small sample size, poor homogeneity, or general measurement tools (scales), rather than a domain-specific assessment, due to lack of specificity.

This is a prospective cohort study, aiming to assess the cognitive and emotional state of DTC patients treated with TSH suppression after thyroidectomy, and find out the specific cognitive impairment and affective disorders associated with TSH suppression therapy.

## Patients and methods

### Ethics

This study conformed to the Declaration of Helsinki regarding ethical principles for medical research in humans. It was approved by the biomedical ethics committee of Inner Mongolia Medical University (No. YKD2014063).

### Trial registration

ClinicalTrials.gov Protocol Registration System: ClinicalTrials.gov ID NCT0266532, Registered on 21 June 2016.

### Clinical characteristics

**Fig. 1:**
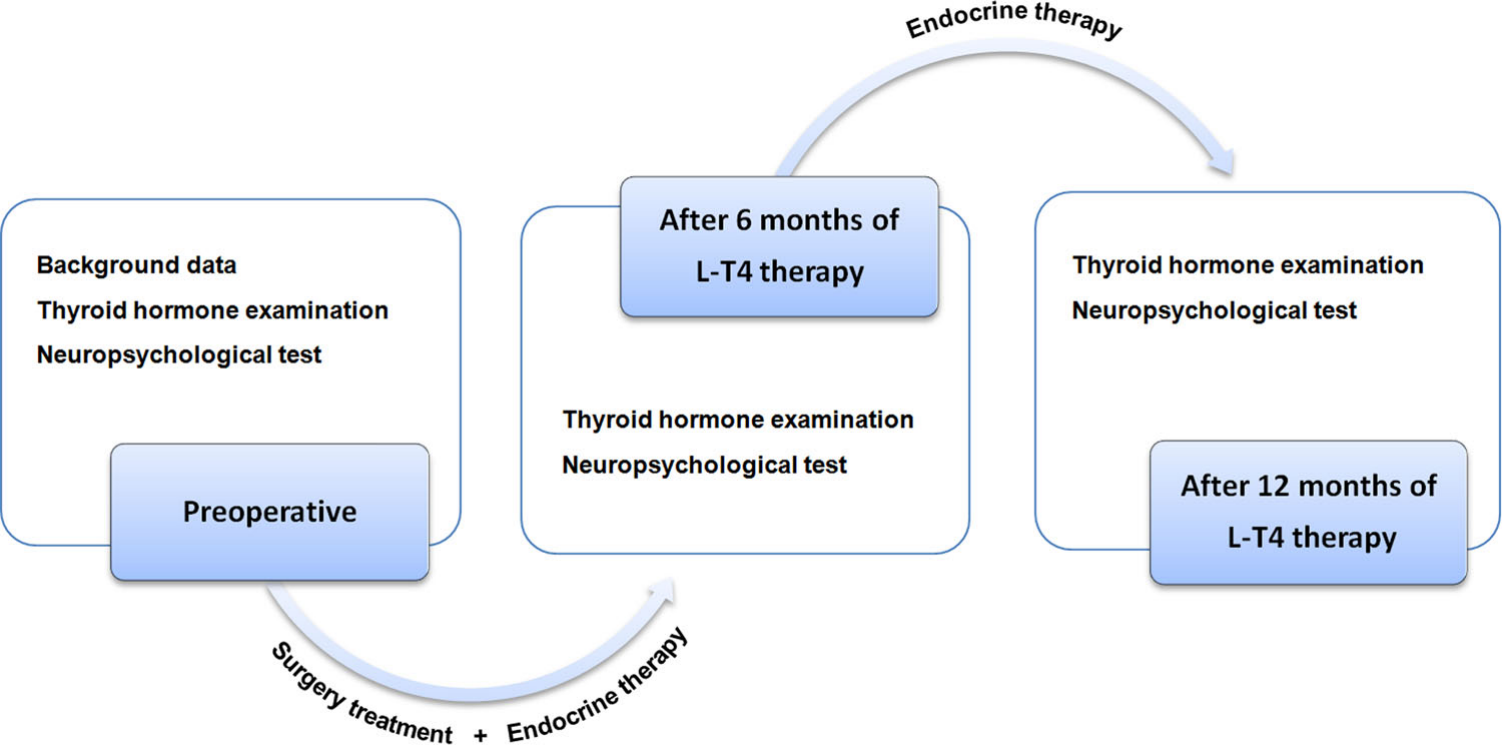
Overview of the research design and experimental process, a prospective longitudinal cohort study designed to examine the impact of thyroid stimulating hormone suppression therapy for differentiated thyroid carcinoma on cognitive functioning. Surgery treatment = lobectomy + central lymph node dissection or total thyroidectomy + central lymph node dissection; endocrine therapy = TSH suppression therapy

Patients diagnosed with DTC and benign thyroid nodules by surgery and pathology, aged between 18 and 65 years, who were good at Chinese, and underwent thyroidectomy with subsequent oral l-T4 replacement therapy from June 2016 to Sept 2018 were included in this study. Surgical treatments in selected patients were completed by the same medical group. Neuropsychological test was performed by two experienced neuro physicians in a standard psychometric room between 8:00 am and 12:00 A.M. to achieve the goal of homogenization of the subjects. Figure 1 shows the research design and experimental process. Entry conditions were as follows: normal control group (NC group): healthy people who underwent physical examination in hospital, and at the same time met the following inclusion criteria: (1) Patient without a history of thyroid disease, results of laboratory examinations: serum T3, T4, FT3, FT4, TSH levels were within the normal range; (2) patient’s age >18 and <65 years, no limitation on gender, his/her level of education ≥6 years; right-handed patient with normal abilities of listening, speaking, reading, writing, as well as language expression and understanding; (4) patient with normal vision or corrected visual acuity; (5) at present or before, patient without diseases of central nervous system or medical behavior that affected cognition; (6) patient without a history of organic brain lesions, a history of long-term loss of consciousness from brain trauma, no epilepsy, dementia, and loss of learning ability; (7) patient without a history of neuropsychiatry, alcohol, and drug abuse; (8) patient without previous diagnosis of cancer, or patient did not underwent radiotherapy and chemotherapy before; (9) patient without other endocrine and autoimmune diseases, and pregnant and lactating women were excluded; and (10) patient who cooperated to complete the test. Thyroxine replacement therapy group (TR group): patient who underwent surgery for benign thyroid nodules in hospital, and at the same time met the following inclusion criteria: (1) surgery, postoperative review, and experimental tests were completed by the same medical group; (2) according to the Chinese version of “Diagnosis and Treatment Guidelines for Thyroid Nodules and Differentiated Thyroid Carcinoma” (first edition) [12], surgical indications, and standardized surgical treatment were followed; (3) patient took l-T4 orally after surgery to complete thyroxine replacement therapy; (4) other inclusion criteria were as same as those in the control group. TSH suppression therapy group (TS group): patient who underwent surgery for DTC, and at the same time in hospital met the following inclusion criteria: (1) surgery, postoperative review and experimental tests were conducted by the same medical group; (2) according to the Chinese version of “Diagnosis and Treatment Guidelines for Thyroid Nodules and Differentiated Thyroid Carcinoma” (first edition) [12], standardized surgical treatment were followed; (3) after surgery, patient took l-T4 orally to complete TSH suppression therapy, the serum FT3 level was normal, FT4 level was slightly elevated or normal, and TSH level was decreased; (4) other inclusion criteria were as same as those in the control group.

### Assessments and outcomes

#### Background data

Clinical data, such as age, gender, level of education, history of diseases, surgical methods, and so on, were consulted in detail.

#### Laboratory examinations

The baseline data of serum thyroid hormone levels of all patients in each group were obtained before surgery. Patients in the TR and TS groups were treated with L-T4 after surgery. The serum thyroid hormone levels were reexamined every 4 weeks to determine that they met the criteria of thyroxine replacement therapy and TSH suppression therapy, and duration of neuropsychological test was accordingly calculated. Neuropsychological test was performed after 6 and 12 months of L-T4 therapy. If serum thyroid hormone levels fluctuated during the period of reexamination, the L-T4 dose was re-adjusted, and duration of neuropsychological test was re-determined.

#### Neuropsychological test

The specific objectives of neuropsychological test for assessing cognitive function and emotional state are shown in Table 1.

**Table 1:** Neuropsychological scale and its implications

Test/measure	Subproject	Neuropsychological domain
MMSE		Global cognition
Clock drawing test		Visuospatial function
Digit symbol substitution test		Information processing speed
Wechsler memory scale (Chinese version)	Personal history	
	Temporal and spatial orientation	Long-term memory
	Digit order relation	
	Visual object recognition	
	Picture recall	
	Visual recall	Short-term memory
	Associative learning	
	Comprehension memory	
	Digit span forward	Attention
	Digit span backward	Working memory
Aphasia battery of Chinese	Confrontation naming	
	Color naming	Naming
	Listing the names	
PHQ-9 test		Depression

Mini-mental state examination (MMSE) was used to assess general cognitive function, including orientation, memory, attention, computational ability, language ability, understanding, copying, etc. The full score was 30 points [13].

Clock drawing test was used to assess visuospatial function. Drawing a closed disc (clock dial): 1 point; no missing of 12 numbers on the clock dial: 1 point; correctly marking the positions of minute hand and hour hand: 1 point. The full score was 4 points [14].

Digit symbol substitution test (DSST) was undertaken to assess information processing speed. Subjects were asked to convert a set of numbers into corresponding symbols, in which the number of correct conversions completed within 90 s was recorded [15].

Chinese version of Wechsler Memory Scale (WMS) was used to assess memory. The content of the test included long-term memory test: (1) personal history (five questions about subject’s personal experience were answered by the subjects, the answers were recorded, without time limit, and the questions could be repeated, but no prompt for answers); (2) temporal and spatial orientation (five questions about the orientation of time and space); (3) digit order relation (three items, ranging from 1 to 100 forward, from 100 to 1 backward, and accumulation). Short-term memory test: (1) visual object recognition (there were eight contents in the Card set A, including graphics, characters, and symbols, take them away after half a min, and subjects had to find eight contents of graphics, characters, and symbols that they have seen from 28 contents available in Card set B); (2) picture recall (let subjects look at 20 familiar pictures for one and a half min, then take the pictures away, and ask subjects to recall; (3) visual recall (let subjects carefully look at the picture on the card, then take the picture away after 10 s, and ask subjects to draw it silently); (4) associative learning (10 pairs of words were presented on a card and read them to subjects, after 5 s, read the previous word of each pair of words, and ask subjects to say the latter word); (5) comprehension memory (read a story to subjects, in order to make it easy for subjects to understand, show the subjects the story written on the cards at the same time, then ask subjects to repeat the story immediately after finishing, and subjects’ recall contents were counted). Digit span test: digit span forward and backward tests (read random numbers with two to nine digits to subjects, then ask them to recite forward and backward numbers, respectively, recite forward numbers from three digits to nine digits, and recite backward numbers from two digits to eight digits) [15, 16].

Aphasia battery of Chinese (ABC) was used to assess naming difficulties. The content of naming test was included as follows: (1) confrontation naming (show objects or pictures in stages, and ask subjects what they are); (2) color naming test (ask questions firstly, and let subjects respond to the correct color); (3) listing the names (ask subjects to say as many vegetable or animal names as possible in a minute) [17, 18].

Patient Health Questionnaire-9 (PHQ-9) depression screening scale was used to assess emotional state. According to the 9 criteria of diagnosing major depressive disorder in the Diagnostic and Statistical Manual of Mental Disorders (fourth edition), the higher the score, the higher the level of depression [19].

### Statistical analysis

In this study, SPSS 19.0 software (IBM, Armonk, NY, USA) was used for statistical analysis. Measurement data were expressed by $\bar x$ ± *s*. Single-factor analysis of variance was used for making comparison among the groups. Least significant difference and *t* test were used for intra-group comparisons. Chi-squared test was employed for comparing enumeration data. *P* value < 0.05 was considered statistically significant.

## Results

### Clinical outcomes of subjects among the three groups

Here, 255 patients were initially included, and 212 patients (83.13%) were screened strictly according to the criteria of enrollment. The main reasons for excluding them from the study were cerebrovascular diseases, other system tumors, incomplete tests and refusal to continue to participate in the study. Of the 212 patients, 33 cases were enrolled in the Normal control group (NC group), including 4 males and 29 females, with an average age of 43.76 years; 69 cases were enrolled in the Thyroxine replacement therapy group (TR group), involving 14 males and 55 females, with an average age of 43.52 years, 64 cases underwent thyroid lobectomy, and 5 cases underwent total thyroidectomy, in which the postoperative pathology was thyroid adenoma or nodular goiter; 110 cases were enrolled in the TSH suppression therapy group (TS group), including 20 males and 90 females, with an average age of 43.44 years, 59 cases underwent thyroid lobectomy and isthmectomy, and 51 cases underwent total thyroidectomy, in which the type of pathology was papillary thyroid cancer. All patients underwent central lymph node dissection of the affected side at the same time. Of the 51 patients who underwent total thyroidectomy, 12 cases received ^131^I treatment. There were no significant differences in terms of age, gender, level of education, serum thyroid hormone levels, as well as the scores of MMSE, clock drawing test, digit symbol substitution test, personal history, temporal and spatial orientation, digit order relation, visual object recognition, picture recall, visual recall, associative learning, comprehension memory, digit span forward, digit span backward, confrontation naming, color naming, and listing the names among the three groups (*P* > 0.05) (Table 2). The PHQ-9 scores in the TS and TR groups were significantly higher than those in the NC group (*P* < 0.01), and the PHQ-9 score in the TS group was also significantly higher than that in the TR group (*P* < 0.01).

**Table 2:** Baseline data of neuropsychological tests in the three groups

Test/measure	NC group	TR group	TS group	*F*(*x*²)	*P* value
Age	43.76 ± 10.5	43.52 ± 10.42	43.44 ± 9.16	0.01	0.99
Gender (male/female)	4/29	14/55	20/90	1.02	0.60^a^
Education level (year)	10.94 ± 3.99	10.35 ± 3.46	11.03 ± 3.71	0.75	0.47
T3	1.1 ± 0.17	1.19 ± 0.17	1.15 ± 0.23	2.30	0.10
T4	8.01 ± 1.74	7.69 ± 1.08	8.12 ± 1.68	1.69	0.19
FT3	3.1 ± 0.33	3.22 ± 0.32	3.1 ± 0.48	1.95	0.14
FT4	1.23 ± 0.2	1.24 ± 0.17	1.25 ± 0.21	0.19	0.82
TSH	2.9 ± 1.88	2.7 ± 1.57	3.11 ± 3.17	0.56	0.57
MMSE	29.12 ± 1.19	29.17 ± 1.19	29.23 ± 1.01	0.13	0.87
Clock drawing test	3.95 ± 0.19	3.88 ± 0.37	3.91 ± 0.37	0.46	0.63
Digit symbol substitution test	48.24 ± 9.65	49.51 ± 8.93	47.2 ± 9.59	1.28	0.28
Personal history	4.58 ± 0.71	4.49 ± 0.72	4.54 ± 0.7	0.17	0.85
Temporal and spatial orientation	5 ± 0	4.96 ± 0.21	4.98 ± 0.13	1.06	0.35
Digit order relation (1 → 100)	58.33 ± 8.9	57.49 ± 6.13	58.58 ± 5.95	0.60	0.55
Digit order relation (100 → 1)	191.52 ± 34.58	190.22 ± 33.32	191.9 ± 29.59	0.06	0.94
Digit order relation (accumulation)	90.09 ± 12.47	88.75 ± 13.01	90.11 ± 12.12	0.27	0.76
Visual object recognition	15.64 ± 0.78	15.27 ± 1.02	15.42 ± 1.12	1.42	0.24
Picture recall	16.85 ± 2.03	16.09 ± 2.22	16.45 ± 2.11	1.47	0.23
Visual recall	11.21 ± 2.79	10.46 ± 2.17	11.28 ± 2.13	2.95	0.05
Associative learning	17.35 ± 3.01	16.51 ± 3.4	16.51 ± 4.1	0.70	0.50
Comprehension memory	10.26 ± 2.71	9.78 ± 1.78	10.16 ± 2.3	0.81	0.45
Digit span forward	8.36 ± 1.29	8.2 ± 1.24	8.14 ± 1.22	0.43	0.65
Digit span backward	5.7 ± 1.1	5.38 ± 1.04	5.57 ± 1.23	1.03	0.36
Confrontation naming	59.27 ± 0.78	59.36 ± 0.78	59.4 ± 0.8	0.31	0.73
Color naming	20 ± 0	19.93 ± 0.6	19.98 ± 0.13	0.66	0.52
listing the names	19.18 ± 1.21	18.58 ± 1.59	19.05 ± 1.64	2.49	0.09
PHQ-9	1.45 ± 1.68	2.39 ± 2.33*	3.38 ± 3.17**	7.28	0.00

^a^Chi-squared test was employed for comparing gender data. Normal T3 level: 0.8–2.0 ng/ml; normal T4 level: 5.1–14.1 mμg/dl; normal FT3 level: 2.0–4.4 pg/mL; normal FT4 level: 0.93–1.7 ng/dL; normal TSH level: 0.2–4.2 μIU/mL

^*^P < 0.01 vs. NC group

^**^P < 0.01 vs. NC group and TR group

### Neuropsychological test results of patients after 6 months of TSH suppression therapy and thyroxine replacement therapy

After 6 months of TSH suppression therapy and thyroxine replacement therapy, there was no significant difference in serum T3 level among the three groups (*P* > 0.05), and no significant differences in serum T4, FT3, FT4, TSH levels between the TR and NC groups (*P* > 0.05), however, the serum T4, FT3, FT4 levels in the TS group were significantly higher than those in the NC and TR groups, while the TSH level was significantly lower than those in the NC and TR groups (*P* < 0.01). There were also no significant differences in the scores of MMSE, clock drawing test, digit symbol substitution test, personal history, temporal and spatial orientation, digit order relation, visual object recognition, associative learning, and color naming among the three groups (*P* > 0.05); the scores of picture recall, visual recall, comprehension memory, and digit span forward in the TS and TR groups were remarkably lower than those in the NC group (*P* < 0.01); the score of digit span backward in the TR group was notably lower than those in the NC and TS groups (*P* < 0.01) The PHQ-9 score in the TS and TR groups was significantly higher than in the NC group (*P* < 0.01); compared with preoperative results, the PHQ-9 score in the TR group was increased, whereas that was decreased in the TS group (Table 3).

**Table 3:** Neuropsychological test results of subjects after 6 months of TSH suppression therapy and thyroxine replacement therapy

Test/Measure	NC group	TR group	TS group	*F*	*P* value
T3	1.1 ± 0.17	1.14 ± 0.17	1.19 ± 0.21	2.95	0.05
T4	8.01 ± 1.74	7.66 ± 1.11	10.15 ± 1.54*	71.86	0.00
FT3	3.1 ± 0.33	3.08 ± 0.49	3.66 ± 0.51*	36.50	0.00
FT4	1.23 ± 0.2	1.15 ± 0.18	1.7 ± 0.37*	80.88	0.00
TSH	2.9 ± 1.88	3.38 ± 1.41	0.33 ± 0.36*	177.95	0.00
MMSE	29.12 ± 1.19	29.25 ± 0.88	29.01 ± 1.29	0.90	0.41
Clock drawing test	3.95 ± 0.19	3.78 ± 0.51	3.89 ± 0.41	2.24	0.11
Digit symbol substitution test	48.24 ± 9.65	44.65 ± 9.53	46.39 ± 10.14	1.57	0.21
Personal history	4.58 ± 0.71	4.57 ± 0.61	4.54 ± 0.62	0.07	0.93
Temporal and spatial orientation	5 ± 0	4.93 ± 0.26	4.96 ± 0.23	1.25	0.29
Digit order relation (1 → 100)	58.33 ± 8.9	57.41 ± 5.45	57.83 ± 5.9	0.25	0.78
Digit order relation (100 → 1)	191.52 ± 34.58	190.28 ± 29.97	190.21 ± 31.69	0.02	0.98
Digit order relation (accumulation)	90.09 ± 12.47	88.46 ± 11.14	90.03 ± 11.22	0.45	0.64
Visual object recognition	15.64 ± 0.78	15.2 ± 1.06	15.21 ± 1.14	2.25	0.11
Picture recall	16.85 ± 2.03	13.97 ± 2.35**	13.98 ± 2.61*	19.26	0.00
Visual recall	11.21 ± 2.79	8.58 ± 2.63**	8.73 ± 2.71*	12.45	0.00
Associative learning	17.35 ± 3.01	16.21 ± 3.11	16.27 ± 3.98	1.32	0.27
Comprehension memory	10.26 ± 2.71	7.55 ± 2.3**	7.47 ± 2.62*	16.51	0.00
Digit span forward	8.36 ± 1.29	7.13 ± 1.24**	7.87 ± 1.15*	13.92	0.00
Digit span backward	5.7 ± 1.1	4.43 ± 1.01**	5.33 ± 1.29	17.40	0.00
Confrontation naming	59.27 ± 0.78	59.41 ± 0.81	58.75 ± 1.14*	10.48	0.00
Color naming	20 ± 0	19.91 ± 0.51	19.97 ± 0.16	1.13	0.32
Listing the names	19.18 ± 1.21	17.64 ± 2.17**	16.05 ± 2.91*	23.04	0.00
PHQ-9	1.45 ± 1.68	3.88 ± 2.89**	2.85 ± 2.86*	9.11	0.00

^*^P < 0.01 vs. NC group and TR group

^**^P < 0.01 vs. NC group

### Neuropsychological test results of patients after 12 months of TSH suppression therapy and thyroxine replacement therapy

After 12 months of TSH suppression therapy and thyroxine replacement therapy, there was no significant difference in serum T3 level among the three groups (*P* > 0.05), and no significant differences in serum T4, FT3, FT4, TSH levels between the TR and NC groups (*P* > 0.05), however, the serum T4, FT3, FT4 levels in the TS group were significantly higher than those in the NC and TR groups, while the serum TSH level was remarkably lower than those in the NC and TR groups (*P* < 0.01). There were no significant differences in the scores of MMSE, clock drawing test, digit symbol substitution test, personal history, temporal and spatial orientation, digit order relation, associative learning, and color naming among the three groups (*P* > 0.05); the scores of visual object recognition, picture recall, visual recall, comprehension memory, and digit span forward in the TS and TR groups were significantly lower than those in the NC group (*P* < 0.01); the score of digit span backward in the TR group was notably lower than that in the NC and TS groups (*P* < 0.01); the scores of confrontation naming and listing the names in the TS group were considerably lower than those in the NC and TR groups (*P* < 0.01). The PHQ-9 score in the TR group was significantly higher than that in the NC group (*P* < 0.01), while no significant difference was found between the TS and NC groups (*P* > 0.05). Compared with preoperative results, the PHQ-9 score in the TR group continued to increase, whereas that continued to decrease in the TS group (Table 4).

**Table 4:** Neuropsychological test results of subjects after 12 months of TSH suppression therapy and thyroxine replacement therapy

Test/measure	NC group	TR group	TS group	*F*	*P* value
T3	1.1 ± 0.17	1.17 ± 0.17	1.17 ± 0.19	1.88	0.16
T4	8.01 ± 1.74	7.74 ± 1.13	10.32 ± 1.39*	87.1	0.00
FT3	3.1 ± 0.33	3.18 ± 0.43	3.63 ± 0.49*	29.86	0.00
FT4	1.23 ± 0.2	1.2 ± 0.19	1.71 ± 0.3*	100.31	0.00
TSH	2.9 ± 1.88	3.18 ± 1.18	0.29 ± 0.27*	202.19	0.00
MMSE	29.12 ± 1.19	29.36 ± 0.87	29.18 ± 1.18	0.78	0.46
Clock drawing test	3.95 ± 0.19	3.8 ± 0.47	3.85 ± 0.38	1.81	0.17
Digit symbol substitution test	48.24 ± 9.65	45.39 ± 9.37	47.15 ± 9.81	1.17	0.31
Personal history	4.58 ± 0.71	4.59 ± 0.55	4.49 ± 0.66	0.63	0.53
Temporal and spatial orientation	5 ± 0	4.96 ± 0.21	4.97 ± 0.16	0.77	0.47
Digit order relation (1 → 100)	58.33 ± 8.9	57.75 ± 5.21	58.01 ± 6.22	0.09	0.91
Digit order relation (100 → 1)	191.52 ± 34.58	190.65 ± 29.57	189.36 ± 33.41	0.07	0.93
Digit order relation (accumulation)	90.09 ± 12.47	89.14 ± 11.06	88.89 ± 11.38	0.14	0.87
Visual object recognition	15.64 ± 0.78	14.99 ± 1.22**	15.13 ± 1.19**	3.67	0.03
Picture recall	16.85 ± 2.03	13.75 ± 2.43**	13.63 ± 2.59**	23.23	0.00
Visual recall	11.21 ± 2.79	8.35 ± 2.06**	8.6 ± 2.55**	17.39	0.00
Associative learning	17.35 ± 3.01	16.25 ± 3.16	16.1 ± 4.09	1.51	0.22
Comprehension memory	10.26 ± 2.71	7.53 ± 2.19**	7.28 ± 2.48**	19.81	0.00
Digit span forward	8.36 ± 1.29	6.94 ± 1.32**	7.86 ± 1.15**	18.75	0.00
Digit span backward	5.7 ± 1.1	4.42 ± 1.01**	5.28 ± 1.23	18.07	0.00
Confrontation naming	59.27 ± 0.78	59.43 ± 0.79	58.4 ± 1.14**	26.01	0.00
Color naming	20 ± 0	19.93 ± 0.49	19.98 ± 0.13	0.94	0.39
listing the names	19.18 ± 1.21	17.8 ± 2.27**	15.24 ± 2.95**	40.58	0.00
PHQ-9	1.45 ± 1.68	4.01 ± 3.2**	2.26 ± 2.91	11.7	0.00

^*^P < 0.01 vs. NC group and TR group

^**^P < 0.01 vs. NC group

### Effect of surgical method on neuropsychological test results in the TS group

In order to determine the effect of thyroidectomy method on cognitive function and emotional state in the TS group, surgical methods were classified into total thyroidectomy and thyroid lobectomy + isthmectomy, and then, intragroup comparison was carried out. Thyroidectomy method had no significant effect on cognitive function and emotional state of DTC patients after 6 and 12 months of TSH suppression therapy (*P* > 0.05) (Supplementary Tables 1 and 2).

### Effect of serum TSH level on neuropsychological test results in the TS group

In order to determine the effect of serum TSH level on cognitive function and emotional state in the TS group, intragroup comparison was performed based on the serum TSH threshold value of 0.5 μIU/mL. Serum TSH level ≤ 0.5 μIU/mL and serum TSH level > 0.5 μIU/mL had no significant effect on cognitive function and emotional state after 6 months of TSH suppression therapy (*P* > 0.05) (Supplementary Table 3). Serum TSH level ≤ 0.5 μIU/mL and serum TSH level > 0.5 μIU/mL had no significant influence on cognitive function after 12 months of TSH suppression therapy (*P* > 0.05), however, when serum TSH level ≤ 0.5 μIU/mL, the PHQ-9 score was significantly higher than that when serum TSH level > 0.5 μIU/mL (*P* < 0.05) (Supplementary Table 4).

### Effect of duration of TSH suppression therapy on neuropsychological test results in the TS Group

According to the above test results, positive items with statistical differences between 6 and 12 months of TSH suppression therapy, as well as preoperative test results were screened out. These positive items were compared by using duration of TSH suppression therapy as the node. It was revealed that there were no significant differences in the scores of visual object recognition, picture recall, visual recall, comprehension memory, digit span forward, and PHQ-9 between 6 and 12 months after TSH suppression therapy (*P* > 0.05), while significant differences were observed in the scores of confrontation naming and listing the names (*P* < 0.01). With the prolongation of TSH suppression therapy, the scores of confrontation naming and listing the names were gradually decreased (Table 5).

**Table 5:** Effect of duration of TSH suppression therapy on neuropsychological test results in the DTC patients

Test/measure	Preoperative	After 6 months	After 12 months	*F*	*P* value
Visual object recognition	15.42 ± 1.12	15.21 ± 1.14	15.13 ± 1.19	1.81	0.16
Picture recall	16.45 ± 2.11	13.98 ± 2.61	13.63 ± 2.59	43.56	0
Visual recall	11.28 ± 2.13	8.73 ± 2.71	8.6 ± 2.55	41.16	0
Comprehension memory	10.16 ± 2.3	7.47 ± 2.62	7.28 ± 2.48	46.67	0
Digit span forward	8.14 ± 1.22	7.87 ± 1.15	7.86 ± 1.15	1.92	0.15
Confrontation naming	59.4 ± 0.8	58.75 ± 1.14	58.4 ± 1.14*	25.89	0
listing the names	19.05 ± 1.64	16.05 ± 2.91	15.24 ± 2.95*	67.28	0
PHQ-9	3.38 ± 3.17	2.85 ± 2.86	2.26 ± 2.91	3.87	0.02

^*^P < 0.01 vs. after 6 months

## Discussion

After thyroidectomy, patients with hypothyroidism may have cognitive impairment, such as attention, executive function, working memory, and emotional impairment (e.g., anxiety and depression) [20, 21]. The thyroid hormone level of patients with hypothyroidism returned to normal after levothyroxine replacement therapy, which could improve their emotional disorder and cognitive ability, while after long-term replacement therapy, there were memory and some emotional impairments [3, 22], indicating that l-T4 replacement therapy cannot fully replace thyroid function. In the Djurovic et al.’s study, 130 Hashimoto’s thyroiditis patients undergoing l-T4 replacement therapy were compared with 111 patients with normal thyroid function, in which the results showed that even if serum thyroid hormone levels returned to normal, the scores of MMSE, TMT-Trail Making Test, phonemic fluency test, and depression in patients undergoing replacement therapy were worsened, and some cognitive functions and emotion impairment were continuously found as well [23]. Similar results were obtained in this study. After 6 and 12 months of l-T4 replacement therapy, were performed in patients (nonmalignant) after they undergoing thyroidectomy, their global cognition function, visuospatial function, long-term memory and naming were not impaired, while their information processing speed, short-term memory, attention, and working memory were impaired, and their depression was aggravated. Bunevicius et al. adopted l-T4 therapy alone or combined with l-T4+T3 replacement therapy to treat patients with hypothyroidism after thyroidectomy, in which the results showed that the combination therapy did not significantly improve cognitive function, however, it could promote the improvement of emotional state [24]. Bononi et al. [25] treated 50 patients (nonmalignant) with combined l-T4+T3 replacement therapy after they underwent thyroidectomy, in which the results showed that the combination therapy can improve the cognitive function and well-being of patients better than the l-T4 replacement therapy alone. The above-mentioned studies suggested that neither l-T4 therapy alone nor the combined l-T4+T3 replacement therapy after thyroidectomy can replace the normal thyroid function. Hogervorst et al. found that high serum FT4 level was associated with cognitive impairment in individuals without thyroid disease, while the possible mechanism was unclear [26]. Medici et al. [27] found that serum TSH level at normal low limit was significantly correlated with depression in elderly people. A survey was conducted in 495 community residents in Korea, and the results showed a significant correlation between low serum TSH level (<0.5) and cognitive impairment [28]. These results confirmed that the serum TSH level in the inhibitory state also leads to cognitive impairment and affective disorder among normal population. After thyroid cancer surgery, although the serum TSH level of patients with subclinical hyperthyroidism treated with TSH suppression therapy is different from that of normal population, long-term TSH suppression therapy can cause cognitive impairment and emotional disorders. Jaracz et al. adopted the Wisconsin Card Sorting Test, Oral Word Association Test, Trail Making Test, Stroop Color-Word Interference Test, and Digit span test in 31 DTC patients who treated with ^131^I and l-T4 suppression after thyroidectomy, in which the results showed that patients’ executive function, information processing speed and attention were significantly decreased in comparison with those in the control group, however, there was no significant difference in Stroop Color-Word Interference test and Digit span test [7]. However, Moon et al. [11] studied 50 elderly DTC patients, and the results showed that long-term TSH suppression therapy had no significant influence on cognitive function, and even for patients with higher serum T4 levels, they had better results in MMSE and Trail Making Test A. Jung et al. [10] studied 90 DTC women, after receiving thyroxine replacement therapy, they showed significant impairment of attention and working memory, and these cognitive impairments were associated with thyroid cancer, age, and level of education. The results of the present study showed that there were both similarities and differences in cognitive impairment between TSH suppression therapy after DTC surgery and l-T4 replacement therapy after thyroidectomy. After TSH suppression therapy after DTC surgery or l-T4 replacement therapy after thyroidectomy was performed, in which global cognitive function, visuospatial function, and long-term memory were not impaired, while short-term memory and attention were impaired. With the prolongation of l-T4 therapy, depression was found in both groups, whereas that was improved in patients treated with TSH suppression after DTC surgery, and that was worsened in patients treated with l-T4 replacement after thyroidectomy. In addition, patients treated with l-T4 replacement after thyroidectomy had impaired working memory, while patients treated with TSH suppression after DTC surgery did not have. The information processing speed of patients treated with TSH suppression after DTC surgery was significantly faster than that of patients treated with l-T4 replacement after thyroidectomy. Furthermore, there were no naming disorders in patients treated with l-T4 replacement after thyroidectomy, while patients treated with TSH suppression after DTC surgery did have, and it was gradually aggravated over time.

Our prospective cohort study found that the global cognitive function, visuospatial function, long-term memory, information processing speed, and working memory were not impaired in patients treated with TSH suppression after DTC surgery, while their short-term memory, attention, and naming were impaired. Despite the existence of depression, the state of depression was gradually improved. We believe that the preoperative diagnosis of thyroid cancer leads to anxiety and psychological distress, however, with the prolongation of therapy after surgery, correct understanding of their own diseases, psychological distress, and depression were improved. To determine whether the residual thyroid tissue has an effect on cognitive impairment after surgery, DTC patients undergoing total thyroidectomy were compared with those undergoing thyroid lobectomy and isthmectomy, and it was revealed that thyroidectomy had no significant impact on cognitive function and emotional state in DTC patients after 6 and 12 months of TSH suppression therapy, indicating that cognitive impairment in DTC patients was associated with TSH suppression therapy. However, after comparing the levels of TSH ≤0.5 μIU/mL and TSH > 0.5 μIU/mL, no evidence was found that excessive TSH suppression therapy can aggravate the degree of cognitive impairment. It is worth noting that with the prolongation of TSH suppression therapy, confrontation naming and listing the names disorders were gradually aggravated, which were not found in other previous studies, perhaps because of the sample size of this study and the specificity of measurement tools. In clinical practice, after DTC surgery, patients treated with TSH suppression therapy often complain that they cannot name a person or an object or use an exact word when they facing them, and they have a feeling of failing to retrieve a word from memory, that is, tip of the tongue phenomenon. They describe that “I know it but I can’t say it”. For example, you cannot say glasses, while you can say “wearing for looking at things”. After receiving word selection hints, patients can choose the correct answer from the nouns listed by examiners and say it accurately. For this reason, ABC was selectively modified in this study, and patients often had prolonged naming reaction time, function word substitution, and circuitous phenomena. Of which, prolongation of naming reaction time was the most direct and common manifestation; function word substitution refers to patients who cannot say the target word, and replace the target word with “that”, “this”, “it”, and so on; circuitous phenomenon refers to the way in which patients typically describe the function, characteristics, use and appearance of the target word to replace the unspeakable target words (make gestures to imitate the function of objects, such as: using forefinger and middle finger as scissors instead of unspeakable scissors). Patients have a typical word selection anomia, which is not a primary memory deficit, however, that is an abstract concept impairment. Patients have not lost words, and they cannot use words in a certain sense, which is also a mild type of fluent aphasia, while it can affect the daily life and social expression of patients, leading to social deficit or social fears, psychological distress, as well as suffering. It is generally believed that the lesion site of this naming disorder was located in the posterior region of the middle temporal gyrus of the dominant cerebral hemisphere or the junction area of the temporal and occipital lobes [29]. Ardila et al. [30] suggested that word selection anomia may be related to the Brodmann area 37 of the brain region. Naming is a complex psychological process, requiring normal visual perception, language perception, flexibility of nervous system and control of word selection, that is, normal naming depends on the synergy of the whole brain. Unfortunately, this study did not perform functional magnetic resonance imaging examinations, thus it was not possible to identify areas of brain function that might be involved. In conclusion, this study revealed that TSH suppression therapy after DTC surgery could lead to short-term memory impairment, attention impairment, word selection anomia, and depression, of which, word selection anomia was aggravated with the prolongation of TSH suppression therapy. These findings indicate that we not only focus on effects of TSH suppression therapy, but also need to manage adverse effects of TSH suppression therapy in DTC patients. Therefore, we suggested that optimal TSH goals for individual patients must balance the potential benefit of TSH suppression therapy with the possible harm from subclinical hyperthyroidism especially in low-risk DTC patients.

## Supplementary information

Supplementary Information.

## Data Availability

Data are available upon request from the authors.

## References

[1] Davis PJ, Zhou M, Davis FB, Lansing L, Mousa SA, Lin HY (2010). Mini-review: cell surface receptor for thyroid hormone and nongenomic regulation of ion fluxes in excitable cells. Physiol. Behav..

[2] Salazar P., Cisternas P., Martinez M., Inestrosa N. C. Hypothyroidism and Cognitive Disorders during Development and Adulthood: Implications in the Central Nervous System. Mol Neurobiol. 2018;2. [Epub ahead of print]. 10.1007/s12035-018-1270-y. 10.1007/s12035-018-1270-y 30073507

[3] Ritchie M, Yeap BB (2015). Thyroid hormone: Influences on mood and cognition in adults. Maturitas.

[4] Siegel RL, Miller KD, Jemal A (2017). Cancer statistics. CA Cancer J. Clin..

[5] Jin S, Yang YT, Bao W (2018). Signaling pathways in thyroid cancer. Vitam. Horm..

[6] Kim HI, Jang HW, Ahn HS, Ahn S, Park SY, Oh YL, Hahn SY, Shin JH, Kim JH, Kim JS, Chung JH, Kim TH, Kim SW, High Serum TSH (2018). Level is associated with progression of papillary thyroid microcarcinoma during active surveillance. J. Clin. Endocrinol. Metab..

[7] Jaracz J, Kucharska A, Rajewska-Rager A, Lacka K (2012). Cognitive functions and mood during chronic thyrotropin-suppressive therapy with L-thyroxine in patients with differentiated thyroid carcinoma. J. Endocrinol. Invest..

[8] Botella-Carretero JI, Galán JM, Caballero C, Sancho J, Escobar-Morreale HF (2003). Quality of life and psychometric functionality in patients with differentiated thyroid carcinoma. Endocr. Relat. Cancer.

[9] Roerink SH, de Ridder M, Prins J, Huijbers A, de Wilt HJ, Marres H, Repping-Wuts H, Stikkelbroeck NM, Timmers HJ, Hermus AR, Netea-Maier RT (2013). High level of distress in long-term survivors of thyroid carcinoma: results of rapid screening using the distress thermometer. Acta Oncol..

[10] Jung MS, Visovatti M (2017). Post-treatment cognitive dysfunction in women treated with thyroidectomy for papillary thyroid carcinoma. Support Care Cancer.

[11] Moon JH, Ahn S, Seo J, Han JW, Kim KM, Choi SH, Lim S, Park YJ, Park DJ, Kim KW, Jang HC (2014). The effect of long-term thyroid-stimulating hormone suppressive therapy on the cognitive function of elderly patients with differentiated thyroid carcinoma. J. Clin. Endocrinol. Metab..

[12] Chinese Medical Association Surgery Society, Chinese Medical Association Endocrine Society (2012). Chinese Management Guidelines for Patients with Thyroid Nodules and Differentiated Thyroid Cancer. Chin. J. Clin. Oncol..

[13] Folstein MF, Folstein SE, McHugh PR (1975). “Mini-mental state”. A practical method for grading the cognitive state of patients for the clinician. J. Psychiatr. Res.

[14] Shulman KI (2000). Clock-drawing: is it the ideal cognitive screening test?. Int J. Geriatr. Psychiatry.

[15] Wechsler D (1981). Adult Intelligence Scale-evised..

[16] Yao S, Chen H, Jiang L, Tam WC (2007). Replication of factor structure of Wechsler Adult Intelligence Scale-III Chinese version in Chinese mainland non-clinical and schizophrenia samples. Psychiatry Clin. Neurosci..

[17] Wu JB, Lyu ZH, Liu XJ, Li HP, Wang Q (2017). Development and standardization of a new cognitive assessment test battery for Chinese aphasic patients: a preliminary study. Chin. Med J..

[18] Cheung RW, Cheung MC, Chan AS (2004). Confrontation naming in Chinese patients with left, right or bilateral brain damage. J. Int Neuropsychol. Soc..

[19] Wang W, Bian Q, Zhao Y, Li X, Wang W, Du J, Zhang G, Zhou Q, Zhao M (2014). Reliability and validity of the Chinese version of the Patient Health Questionnaire (PHQ-9) in the general population. Gen. Hosp. Psychiatry.

[20] Constant EL, Adam S, Seron X, Bruyer R, Seghers A, Daumerie C (2005). Anxiety and depression, attention, and executive functions in hypothyroidism. J. Int Neuropsychol. Soc..

[21] Schraml FV, Goslar PW, Baxter L, Beason-Held LL (2011). Thyroid stimulating hormone and cognition during severe, transient hypothyroidism. Neuro Endocrinol. Lett..

[22] Wekking EM, Appelhof BC, Fliers E, Schene AH, Huyser J, Tijssen JG, Wiersinga WM (2005). Cognitive functioning and well-being in euthyroid patients on thyroxine replacement therapy for primary hypothyroidism. Eur. J. Endocrinol..

[23] Djurovic M, Pereira AM, Smit JWA, Vasovic O, Damjanovic S, Jemuovic Z, Pavlovic D, Miljic D, Pekic S, Stojanovic M, Asanin M, Krljanac G, Petakov M (2018). Cognitive functioning and quality of life in patients with Hashimoto thyroiditis on long-term levothyroxine replacement. Endocrine.

[24] Bunevicius R, Jakuboniene N, Jurkevicius R, Cernicat J, Lasas L, Prange AJ (2002). Thyroxine vs thyroxine plus triiodothyronine in treatment of hypothyroidism after thyroidectomy for Graves’ disease. Endocrine.

[25] Bononi M, De Toma G, Scarpini M, Miccini M, De Cesare A, Meucci M, Amore Bonapasta S, Celotto A, Tocchi A (2010). Hormone replacement therapy after total thyroidectomy. Can the combined treatment be considered effective to get metabolic adequacy? Preliminary results. G Chir..

[26] Hogervorst E, Huppert F, Matthews FE, Brayne C (2008). Thyroid function and cognitive decline in the MRC Cognitive Function and Ageing Study. Psychoneuroendocrinology.

[27] Medici M, Direk N, Visser WE, Korevaar TI, Hofman A, Visser TJ, Tiemeier H, Peeters RP (2014). Thyroid function within the normal range and the risk of depression: a population-based cohort study. J. Clin. Endocrinol. Metab..

[28] Kim JM, Stewart R, Kim SY, Bae KY, Yang SJ, Kim SW, Shin IS, Yoon JS (2010). Thyroid stimulating hormone, cognitive impairment and depression in an older korean population. Psychiatry Investig..

[29] Wilder-Smith E, Taghavi E, Mcconnell H (1994). Anomic (word-selection) aphasia due to inferior—anterior temporal atrophy. J. Aphasiology..

[30] Ardila A, Bernal B, Rosselli M (2015). Language and visual perception associations: meta-analytic connectivity modeling of Brodmann area 37. Behav. Neurol..

