# Supplementary material for: Naming difficulties after thyroid stimulating hormone suppression therapy in patients with differentiated thyroid carcinoma: a prospective cohort study
Source: Endocrine. 2019 May 5;65(2):327–37. doi: 10.1007/s12020-019-01943-8 (PMC6656796; doi:10.1007/s12020-019-01943-8)
Supplement: Supplementary file 1 — Supplementary Information. [file 12020_2019_1943_MOESM1_ESM.docx]

| **Table S1. Effect of surgical method on neuropsychological test results after 6 months of TSH suppression therapy** | | | | |
| --- | --- | --- | --- | --- |
| Test/Measure | Lobectomy + Isthmectomy | Total thyroidectomy | T | P-value |
| MMSE | 28.98±1.36 | 29.04±1.22 | -0.23 | 0.82 |
| Clock drawing test | 3.9±0.3 | 3.88±0.52 | 0.2 | 0.84 |
| Digit symbol substitution test | 47.71±10.16 | 44.86±10 | 1.48 | 0.14 |
| Personal history | 4.54±0.65 | 4.53±0.58 | 0.11 | 0.91 |
| Temporal and spatial orientation | 4.93±0.31 | 5±0 | -1.54 | 0.13 |
| Digit order relation（1→100） | 58.18±4.25 | 57.43±7.39 | 0.66 | 0.51 |
| Digit order relation（100→1） | 187.71±32.96 | 193.1±30.21 | -0.89 | 0.38 |
| Digit order relation（accumulation） | 89.66±10.14 | 90.45±12.45 | -0.37 | 0.71 |
| Visual object recognition | 15.2±1.14 | 15.23±1.14 | -0.1 | 0.92 |
| Picture recall | 13.95±2.73 | 14.02±2.49 | -0.14 | 0.89 |
| Visual recall | 8.71±2.65 | 8.75±2.8 | -0.06 | 0.95 |
| Associative learning | 16.69±3.78 | 15.79±4.18 | 1.18 | 0.24 |
| Comprehension memory | 7.48±2.37 | 7.45±2.91 | 0.06 | 0.95 |
| Digit span forward | 7.92±1 | 7.82±1.31 | 0.42 | 0.68 |
| Digit span backward | 5.44±1.33 | 5.2±1.23 | 0.99 | 0.32 |
| Confrontation naming | 58.69±1.2 | 58.81±1.07 | -0.58 | 0.56 |
| Color naming | 19.98±0.13 | 19.96±0.2 | 0.71 | 0.48 |
| listing the names | 16.25±2.69 | 15.8±3.16 | 0.81 | 0.42 |
| PHQ-9 | 2.71±2.56 | 3±3.19 | -0.52 | 0.60 |

*Note*: * No significant intergroup difference (*P* > 0.05).

| **Table S2. Effect of surgical method on neuropsychological test results after 12 months of TSH suppression therapy** | | | | |
| --- | --- | --- | --- | --- |
| Test/Measure | Lobectomy + Isthmectomy | Total thyroidectomy | T | P-value |
| MMSE | 29.17±1.22 | 29.2±1.15 | -0.12 | 0.91 |
| Clock drawing test | 3.86±0.35 | 3.84±0.42 | 0.29 | 0.77 |
| Digit symbol substitution test | 48.07±10.66 | 46.08±8.72 | 1.06 | 0.29 |
| Personal history | 4.54±0.68 | 4.43±0.64 | 0.88 | 0.38 |
| Temporal and spatial orientation | 4.95±0.22 | 5±0 | -1.64 | 0.10 |
| Digit order relation（1→100） | 58.31±4.4 | 57.67±7.84 | 0.54 | 0.59 |
| Digit order relation（100→1） | 185.31±36.61 | 194.05±28.93 | -1.38 | 0.17 |
| Digit order relation（accumulation） | 88.44±10.64 | 89.41±12.27 | -0.44 | 0.66 |
| Visual object recognition | 15.14±1.03 | 15.13±1.37 | 0.04 | 0.97 |
| Picture recall | 13.53±2.74 | 13.75±2.43 | -0.44 | 0.66 |
| Visual recall | 8.76±2.53 | 8.41±2.58 | 0.72 | 0.47 |
| Associative learning | 16.45±3.87 | 15.71±4.35 | 0.95 | 0.34 |
| Comprehension memory | 7.41±2.36 | 7.14±2.63 | 0.55 | 0.58 |
| Digit span forward | 7.81±1.06 | 7.92±1.25 | -0.49 | 0.62 |
| Digit span backward | 5.34±1.37 | 5.22±1.05 | 0.52 | 0.60 |
| Confrontation naming | 58.39±1.09 | 58.42±1.2 | -0.15 | 0.88 |
| Color naming | 20±0 | 19.96±0.2 | 1.54 | 0.13 |
| listing the names | 15.39±2.88 | 15.06±3.04 | 0.59 | 0.56 |
| PHQ-9 | 2.27±2.55 | 2.25±3.3 | 0.03 | 0.98 |

*Note*: * No significant intergroup difference (*P* > 0.05).

| **Table S3. Effect of serum TSH level on neuropsychological test results after 6 months of TSH suppression therapy** | | | | |
| --- | --- | --- | --- | --- |
| Test/Measure | TSH≤0.5 group | TSH＞0.5 group | T | P-value |
| MMSE | 28.98±1.36 | 29.1±1.08 | -0.46 | 0.65 |
| Clock drawing test | 3.9±0.44 | 3.86±0.35 | 0.44 | 0.66 |
| Digit symbol substitution test | 46.02±10.28 | 47.41±9.82 | -0.63 | 0.53 |
| Personal history | 4.59±0.59 | 4.38±0.68 | 1.61 | 0.11 |
| Temporal and spatial orientation | 4.98±0.22 | 4.93±0.26 | 0.88 | 0.38 |
| Digit order relation（1→100） | 57.72±6.22 | 58.14±4.98 | -0.32 | 0.75 |
| Digit order relation（100→1） | 191.81±29.54 | 185.72±37.25 | 0.89 | 0.38 |
| Digit order relation（accumulation） | 89.84±11.2 | 90.55±11.48 | -0.29 | 0.77 |
| Visual object recognition | 15.2±1.23 | 15.24±0.84 | -0.15 | 0.88 |
| Picture recall | 13.95±2.55 | 14.07±2.79 | -0.21 | 0.83 |
| Visual recall | 8.75±2.68 | 8.66±2.83 | 0.17 | 0.87 |
| Associative learning | 15.94±4.16 | 17.19±3.33 | -1.45 | 0.15 |
| Comprehension memory | 7.41±2.67 | 7.62±2.52 | -0.36 | 0.72 |
| Digit span forward | 7.81±1.07 | 8.03±1.35 | -0.88 | 0.38 |
| Digit span backward | 5.28±1.23 | 5.45±1.45 | -0.59 | 0.56 |
| Confrontation naming | 58.77±1.19 | 58.69±0.99 | 0.31 | 0.76 |
| Color naming | 19.98±0.16 | 19.97±0.19 | 0.28 | 0.78 |
| listing the names | 16.1±3.09 | 15.9±2.4 | 0.32 | 0.75 |
| PHQ-9 | 3.14±3.05 | 2.03±2.08 | 1.8 | 0.08 |

*Note*: * No significant intergroup difference (*P* > 0.05); TSH Unit=μIU/mL

| **Table S4. Effect of serum TSH level on neuropsychological test results after 12 months of TSH suppression therapy** | | | | |
| --- | --- | --- | --- | --- |
| Test/Measure | TSH≤0.5 group | TSH＞0.5 group | T | P-value |
| MMSE | 29.14±1.23 | 29.37±0.9 | -0.75 | 0.45 |
| Clock drawing test | 3.85±0.39 | 3.89±0.32 | -0.51 | 0.61 |
| Digit symbol substitution test | 46.77±9.24 | 48.95±12.34 | -0.88 | 0.38 |
| Personal history | 4.51±0.66 | 4.42±0.69 | 0.51 | 0.61 |
| Temporal and spatial orientation | 4.98±0.15 | 4.95±0.23 | 0.74 | 0.46 |
| Digit order relation（1→100） | 57.74±6.55 | 59.32±4.18 | -1.01 | 0.32 |
| Digit order relation（100→1） | 190.05±28.64 | 186.05±51.41 | 0.47 | 0.64 |
| Digit order relation（accumulation） | 88.9±11.26 | 88.84±12.29 | 0.02 | 0.98 |
| Visual object recognition | 15.15±1.16 | 15.05±1.38 | 0.32 | 0.75 |
| Picture recall | 13.53±2.54 | 14.11±2.85 | -0.88 | 0.38 |
| Visual recall | 8.4±2.53 | 9.58±2.43 | -1.86 | 0.07 |
| Associative learning | 16.07±3.92 | 16.29±4.98 | -0.22 | 0.83 |
| Comprehension memory | 7.1±2.44 | 8.18±2.54 | -1.76 | 0.08 |
| Digit span forward | 7.8±1.13 | 8.16±1.21 | -1.23 | 0.22 |
| Digit span backward | 5.18±1.12 | 5.79±1.58 | -2.01 | 0.05 |
| Confrontation naming | 58.44±1.19 | 58.24±0.87 | 0.7 | 0.48 |
| Color naming | 19.98±0.15 | 20±0 | -0.65 | 0.52 |
| listing the names | 15.08±3.04 | 16±2.38 | -1.25 | 0.22 |
| PHQ-9 | 2.53±3.09 | 1±1.29* | 2.11 | 0.04 |

*Note*: *P＜0.05 *vs.* TSH≤0.5 group; TSH Unit=μIU/mL.
